# Draft Genome Sequence of *Bacillus* sp. Strain IITD106, Isolated from Oil-Contaminated Soil

**DOI:** 10.1128/MRA.00982-21

**Published:** 2021-12-02

**Authors:** Arif Nissar Zargar, Saroj Mishra, Manoj Kumar, Preeti Srivastava

**Affiliations:** a Department of Biochemical Engineering and Biotechnology, Indian Institute of Technology Delhi, New Delhi, India; b Indian Oil Corporation, R&D Centre, Faridabad, India; University of Rochester School of Medicine and Dentistry

## Abstract

Here, we report the whole-genome sequence of *Bacillus* sp. strain IITD106. The bacterium has the unique ability to produce saponins. The complete nucleotide sequence will provide insights into the various genes and regulators involved in the biosynthesis of saponin.

## ANNOUNCEMENT

*Bacillus* sp. strain IITD106 was isolated from crude oil-contaminated soil by enrichment culture using asphaltene as a carbon source (1). The bacterium was found to produce a unique biosurfactant, saponin, which is usually reported from plants (2). Whole-genome sequencing was essential not only to understand the different genes involved in the biosynthesis of saponin but also to explore other catabolic abilities of the bacterium.

For extraction of the genomic DNA, the culture was inoculated into Luria broth and incubated at 30°C for 24 h. Genomic DNA was isolated using the DNeasy PowerSoil Pro kit (catalog number 47016; Qiagen). To determine the whole-genome sequence, a library was prepared using the NEBNext Ultra II FS DNA library preparation kit with sample purification beads (catalog number E6177; New England Biolabs). Briefly, the genomic DNA was enzymatically fragmented by targeting 200–300 bp fragments. End repairing of the fragments was performed to create blunt ends, and adenylation was performed by adding a single A to the 3′ ends. This was followed by ligation with loop adapters and cleavage by the uracil-specific excision reagent provided in the kit. The DNA was purified using AMPure beads and enriched using six cycles with NEBNext Ultra II Q5 master mix, Illumina universal primers, and sample-specific octamer primers. The sequences of the universal Illumina flow cell adapters were as follows: Illumina P5 adaptor, 5′-AATGATACGGCGACCACCGAGATCTACAC-3′; Illumina P7 adaptor, 5′-CAAGCAGAAGACGGCATACGAGAT-3′. The bar code indexes of the octamers were as follows: P7 index, UDI16, AACGCATT; P5 index, UDI16, CAACCTGC.

Paired-end sequencing was carried out using the Illumina HiSeq 4000 platform. Trim Galore v0.6.6 was used with default settings for trimming and removal of the adapter sequences (3). The read length was 150 bp, and raw reads were assembled using Unicycler v0.4.8 with default settings (4). The total number of reads obtained was 7,800,000. The total size of the assembly obtained was 4,486,399 bp, distributed in 143 contigs, and the GC content was 41%. The *N*_50_ value for the assembled contigs was 82,061 bp, and the sequence coverage was 269×.

The genome was annotated using the NCBI Prokaryotic Genome Annotation Pipeline (PGAP) v5.2 (5). A total of 4,385 protein-coding genes and 90 pseudogenes were predicted. The BLAST search results revealed 99.72% identity to Bacillus wuyishanensis (6).

The gene annotation analysis of *Bacillus* sp. strain IITD106 revealed the presence of several genes involved in the biosynthesis of saponin. The antibiotic resistance genes were identified *in silico* using CARD (7). Interestingly, genes for resistance to antibiotics such as doxorubicin, tetracycline, linearmycin, fosmidomycin, bicyclomycin, and tunicamycin were detected. A cluster of *ssuACB* genes, which are responsible for the specific uptake of aliphatic sulfonates, was identified. Genes involved in polyaromatic hydrocarbon degradation, such as catechol 2,3-dioxygenase and the putative ring cleavage extradiol dioxygenase MhqA, were identified in the genome of this organism.

### Data availability.

The whole-genome sequence of *Bacillus* sp. strain IITD106 has been deposited in DDBJ/ENA/GenBank under accession number JAIAZY000000000, BioProject number PRJNA750262, BioSample number SAMN20447436, and SRA accession number SRX12347337.
